# Multistage nucleic acid amplification induced nano-aggregation for 3D hotspots-improved SERS detection of circulating miRNAs

**DOI:** 10.1186/s12951-022-01500-y

**Published:** 2022-06-16

**Authors:** Yudie Sun, La Fang, Yang Yi, Aobo Feng, Kui Zhang, Jing-Juan Xu

**Affiliations:** 1 School of Chemistry and Chemical Engineering, Anhui University of Technology, Ma Xiang Road, Ma ‘anshan, 243032 An-hui People’s Republic of China; 2 State Key Laboratory of Analytical Chemistry for Life Science, School of Chemistry and Chemical Engineering, Nan-Jing University, Nanjing, 210023 People’s Republic of China

**Keywords:** Circulating miRNAs, Nucleic acid amplification, SERS, 3D hotspots, Nano-aggregation

## Abstract

Circulating miRNAs in the blood can regulate disease development and thus indicate disease states via their various expression levels. For these reasons, circulating miRNAs constitute useful biomarkers, and an approach to the accurate detection of circulating miRNAs is attractive in the diagnosis and treatment of diseases. However, methods for clinical detecting of circulating miRNA that take both sensitivity and practicality into account are still needed. Therefore, we aimed herein to solve some inherent problems in the actual detection using a robust surface-enhanced Raman scattering (SERS) platform with integrated nucleic acid amplification and nanoparticle aggregation to construct 3D hotspots for improving performance of analyzing circulating miRNAs. After target recognition and initial signal amplification by DNAzyme, we observed that release triggered an open hairpin DNA on gold nanoparticles (AuNPs), which then promote AuNP aggregation, causing the accumulation of a large number of hotspots in three-dimention. The SERS biosensor achieved a better performance than the sandwich-type separation detection, with a low detection limit (0.37 fM) and a broad linear range (1 fM–10 nM) in liquids. This SERS platform can be used as a powerful tool for the detection of circulating miRNAs, and it can be used to improve the sensitivity and accuracy of various clinical-disease diagnoses.

**Graphical Abstract:**

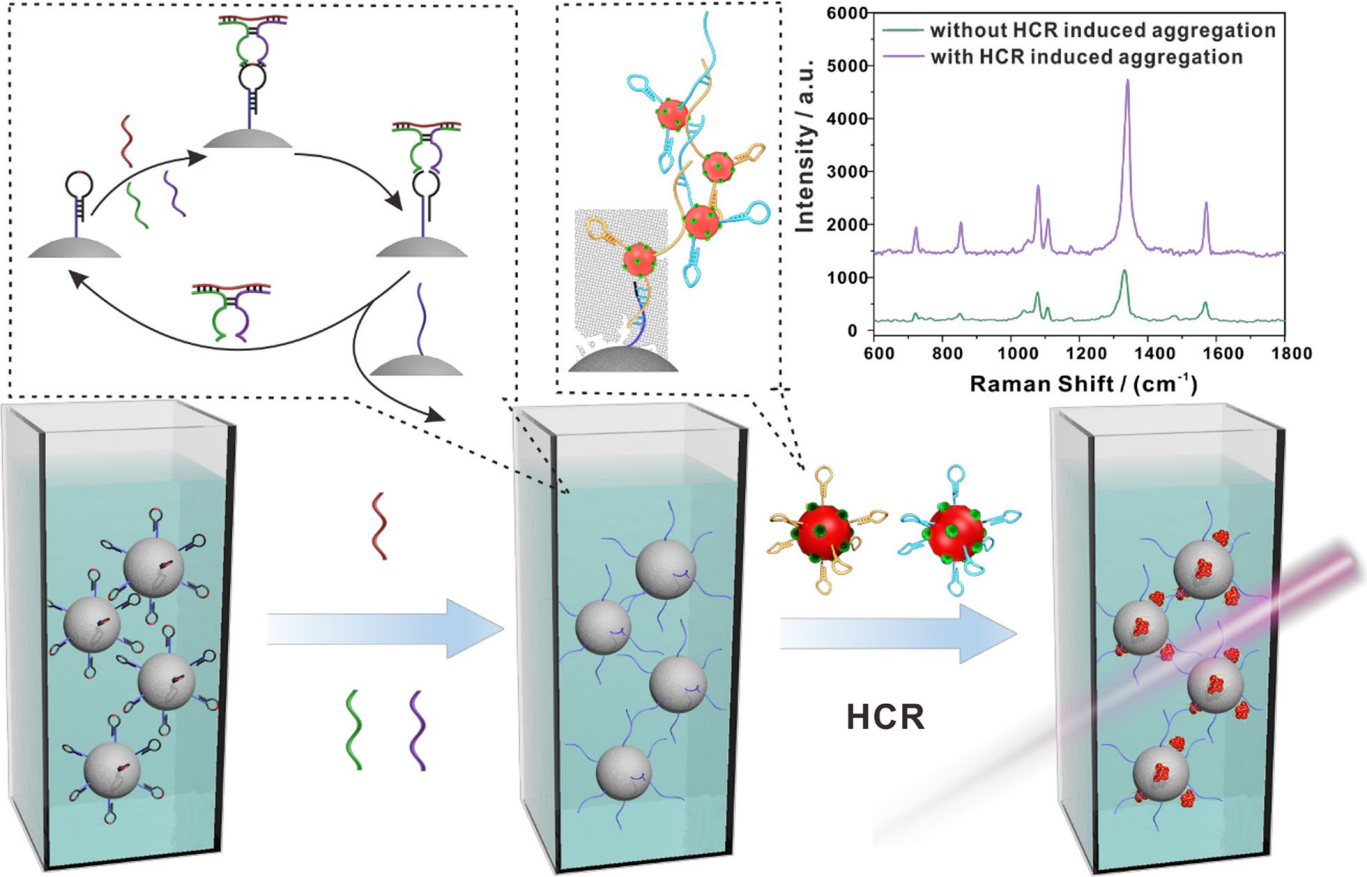

**Supplementary Information:**

The online version contains supplementary material available at 10.1186/s12951-022-01500-y.

## Introduction

Circulating microRNAs (miRNAs) are small non-protein-coding RNAs that are presented in peripheral blood [1]. Some circulating miRNAs participate in gene regulation and are closely involved in diseases such as cancer, other tumorous conditions, and coronary heart disease. The expression levels of circulating miRNAs can vary with disease states and can regulate disease development [2–5]. Reports have shown that circulating miRNAs are useful biomarkers for diseases as their presence in serum is relatively stable [6, 7]. Therefore, accurate detection of disease-related circulating miRNAs is considered an attractive approach for the diagnosis and potentially the treatment of diseases.

A few novel methods based on nucleic acid amplification, including ratio design of electrochemiluminescence assay [8, 9], combinations of logic circuits with fluorescence imaging [10, 11], and MNAzyme-assisted inductively coupled plasma–mass spectrometry detection technology [12], have been used for miRNA detection and intracellular miRNA imaging. However, the expression levels of circulating miRNAs are low, and various types of miRNAs can coexist in circulating blood. In addition, such disruptors in circulating blood are more complex than those in cells. Due to these factors, which produce challenges in developing widely used clinical detecting methods for circulating miRNAs, the available circulating miRNA detection platforms usually complex. Fortunately, surface-enhanced Raman spectroscopy (SERS), which is a fast, sensitive, and nondestructive analytical technique, can possibly be used as a promising approach for qualitative and quantitative analyses of circulating miRNAs in clinical applications [13].

The SERS platform for sensing miRNA has recently shown improved sensitivity that was achieved principally through the synthesis of novel nanomaterials as probes and substrates. However, these methods often require complex material preparation processes [14–17]. Another miRNA detection method that combines nucleic acid amplification technology with nanoprobes or labeled probes has also been reported to detect miRNA. However, these methods require unique probes and are difficult to operate [18–20]. Furthermore, other detection methods that rely on the differences in SERS signals from the different nucleobases are highly susceptible to interference by various substances in the blood [21–23]. The signal generated from agglomeration-free system does not satisfy detection requirements due to low abundance of circulating miRNAs in complex environments, such as blood, and the performance of SERS activity is greatly affected by the number of hot spots presented in plasmonic nanostructures [24–26]. Compared with signals of single nanoparticles, aggregated nanoparticles usually display significantly enhanced SERS signals [27–30]. Therefore, a strategy that can fabricate more hot spots is key to developing a more sensitive and reliable SERS detection platform.

**Scheme 1:**
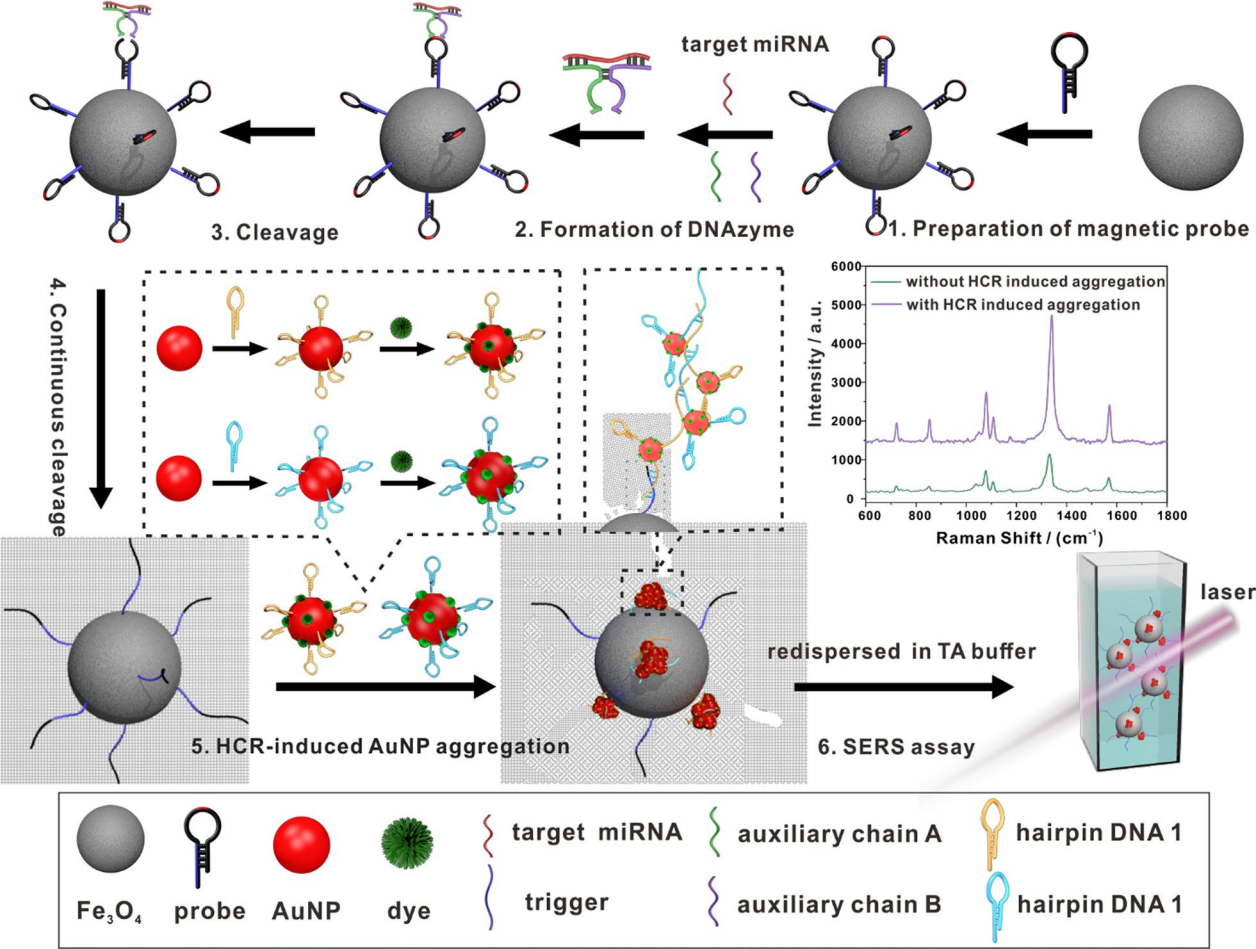
Schematic illustration showing the circulating miRNA detection principle of the SERS platform

Herein, we propose a SERS platform for circulating miRNA detection that integrates nucleic acid amplification with nanoparticle aggregation. The focus of our studies is on miRNA-499, miRNA-328, and miRNA-208, because the levels of these miRNAs are robustly increased in the blood circulation after myocardial infarction [31, 32]. Accurate identification and efficient capture of miRNAs were achieved using an assembled DNAzyme structure that we have reported previously [33]. The principle of the SERS platform in detecting circulating miRNA is shown in Scheme 1. First, hairpin DNA probes containing rA sites were modified on magnetic beads through functional amino groups. Then, in the presence of target miRNA, the DNAzyme component structure that can cut off the rA sites can be formed with the help of auxiliary chain A (DA) and auxiliary chain B (DB). In this way, target recognition and continuous cleavage take place on the surface of the magnetic bead. Subsequently, the cleavage product on the magnetic bead can open the hairpin on the gold nanotag and then trigger a hybridization chain reaction (HCR). As shown in the inset of Scheme 1, the opened hairpin DNA 1 (hp1) on one gold nanotag opens a hairpin DNA 2 (hp2) on the other gold nanotag; the opened hp2 then opens another hp1, and the gold nanometers are pulled together. The same process is then repeated, causing additional gold nanoparticles to be drawn together. The integration of HCR amplification with nanoparticle aggregation increases the generation of hot spots, which can then greatly improve SERS performance. Because there are many hairpin DNA molecules on one gold nanotag, the AuNPs are not assembled in a straight line, but they are instead clustered together. Finally, unreacted gold nanotag are washed away by magnetic separation, and the aggregates on the magnetic nanoprobe are redispersed in TA buffer before being subjected to liquid SERS measurements [34, 35]. Because this novel SERS sensor embeds nucleic acid amplification into the structure of plasmonic hot spots, its sensitivity and reproducibility are greatly improved. Owing to its universal magnetic probe and assembled DNAzyme structure, this sensing platform can be applied to detect any miRNAs, and it may have implications in the development of high-throughput clinical diagnostic strategies [36, 37].

## Materials and methods

### Materials and chemicals

All the DNA used in this study was purchased from Kangbei Biotechnology Co. Ltd. (Ningbo, China), and sequences of all the designed nucleic acid structures are listed in Additional file 1: Table S1. Tris, polyacrylamide, and agarose were obtained from Sangon Biotechnology Co. Ltd. (Shanghai, China). Chloroauric acid (HAuCl_4_), ferric trichloride (FeCl_3_), magnesium acetate (MgAC_2_), 4-nitrobenzenethiol, Tris (2-carboxyethyl) phosphine hydrochloride (TCEP) and all of the other analytical grade reagents were purchased from Sinopharm Chemical Reagent Co. Ltd. (Shanghai, China). Serum was obtained from Ma’anshan First People's Hospital (Ma’anshan, China).

### Exploring multistage nucleic acid amplification

Relevant nucleic acid structures were designed based on our previous work [19, 33, 38, 39]. Characterization of target recognition and the first amplification step with DNAzyme were performed using polyacrylamide gel electrophoresis (10% w/w gel) after incubating the samples at 37 °C for 9 h (10 mM MgAC_2_, TE buffer). The modified hairpin structures of HCR and the HCR results were verified by polyacrylamide gel electrophoresis (6% w/w gel). Both polyacrylamide gel electrophoretic assays were carried out at 96 V for 4 h (25 °C).

### Preparation of magnetic biological probe (MBP)

Carboxyl-modified Fe_3_O_4_ nanoparticles were synthesized according to the method described in the literature [40]. In brief, FeCl_3_ (0.6 g) was mixed with trisodium citrate (0.4 g) in ethylene glycol and stirred for 30 min. Then, 1.2 g of sodium acetate was added into the mixed solution and stirred until dissolved completely. The mixture was then placed in an oven at 230 °C for 12 h. Finally, the obtained Fe_3_O_4_ nanoparticles were washed with ethanol and water.

The carboxyl groups on the surface of Fe_3_O_4_ nanoparticles were first activated with EDC and sulfo-NHS. After that the surface of Fe_3_O_4_ nanoparticles were modified with aminated hairpin DNA containing rA sites through peptide bonds. An aliquot (500 μL) of 0.2 mg/mL Fe_3_O_4_ were incubated with 100 μL of 10 μM aminated probe DNA in 1 mL of PBS buffer (pH 7.4) at room temperature. After sealing with NH_2_-PEG, the obtained magnetic biological probes were washed three times with PBS buffer and then dispersed in 500 μL of PBS buffer.

### Preparation of gold nanotag

The gold nanotag was prepared by assembling HCR hairpin units and Raman dyes on the surface of AuNP. First, AuNPs with a size of 16 nm were synthesized by reducing chloroauric acid with citric acid [41, 42]. Then, the TCEP treated HCR hairpin units were modified onto the surface of AuNP using the salt aging method. In detail, 6 μL of 10 μM treated hairpin DNA was mixed with 10 μL of 10 nM AuNPs. The mixture was then incubated in a buffer containing 0.05 M sodium citrate and 0.01% Tween-20 for 3 h. Subsequently, 5 μL of 3 M NaCl was added to the above solution every 5 min until its final concentration reached 0.3 M. Next, 1 mM Raman dye (4-nitrophenylthiophenol) was added to the solution and incubated for 30 min. Finally, unbound DNA and excess dyes were washed, and the prepared nanotags were stored in TE buffer.

### Induction of AuNP Aggregation by HCR

Two corresponding gold nanotags used for HCR reaction were mixed in 10 mM TE buffer containing 10 mM MgAc_2_. Then, 1 nM trigger DNA was added to the mixture and incubated for 3 h at 37 °C. The products were then characterized by dynamic light scattering (DLS) and UV—vis spectroscopy. After centrifugation, the products were further characterized by agarose electrophoresis (3% w/w gel) in TBE buffer at 64 V for 2 h (25 °C). The final composite state on the magnetically separated substrate was characterized by a transmission electron microscope (TEM) (JEM-2100).

### Detection of circulating miRNA by the SERS platform

To promote the formation of DNAzyme, 90 μL of 0.2 mg/mL MBP was mixed with 100 nM DNAzyme auxiliary strand A (DA) and 100 nM auxiliary strand B (DB). Then, 10 μL of the target miRNA was added and incubated with 10 mM MgAc_2_ at 37 °C for 9 h to allow target recognition and signal amplification. For the detection of circulating miRNA in serum, 1 μL of the target miRNA was added into 10 μL of patients’ serum to achieve simulation samples with concentrations ranging from 1 fM to 1 nM. Then the simulation sample was added into 90 μL of the mixture of MBP, DA, and DB, and incubated with 10 mM MgAc_2_ at 37 °C for 9 h. For SERS measurement, 1 nM each of two gold nanotags was added to the above solution and incubated with the DNAzyme cleavage product at 37 °C for 2.5 h. Finally, a large number of AuNP aggregates were allowed to grow on the surface of the magnetic biological probe, which greatly contributed to the SERS signal. After excess gold nanotags were washed away, the products were redispersed in 100 μL of 10 mM TA buffer before subjecting them to liquid SERS measurements. Raman measurements were conducted on a portable Raman system (BWTEK, USA) equipped with an enhanced Raman cuvette holder and a 785 nm laser [23]. To reduce the volume of samples, a 100 μL custom-made cuvette was used in the liquid SERS assay (the custom-made cuvette and detection device are depicted in Additional file 1: Figure S1). The SERS signal was collected at a laser power of 30 mW, with an exposure time of 10 s.

## Results and discussion

### Characterization of the SERS platform with integrated nucleic acid amplification and nano-aggregation.

According to the electromagnetic field enhancement mechanism of SERS, we designed a multi-hot spot SERS biosensor for circulating miRNA detection by combing nucleic acid amplification with nano-aggregation. The designed SERS platform exhibited improved performance compared with that of the agglomeration-free separation detection, and multiple circulating miRNAs associated with myocardial infarction could be detected with high sensitivity [27].

**Fig. 1:**
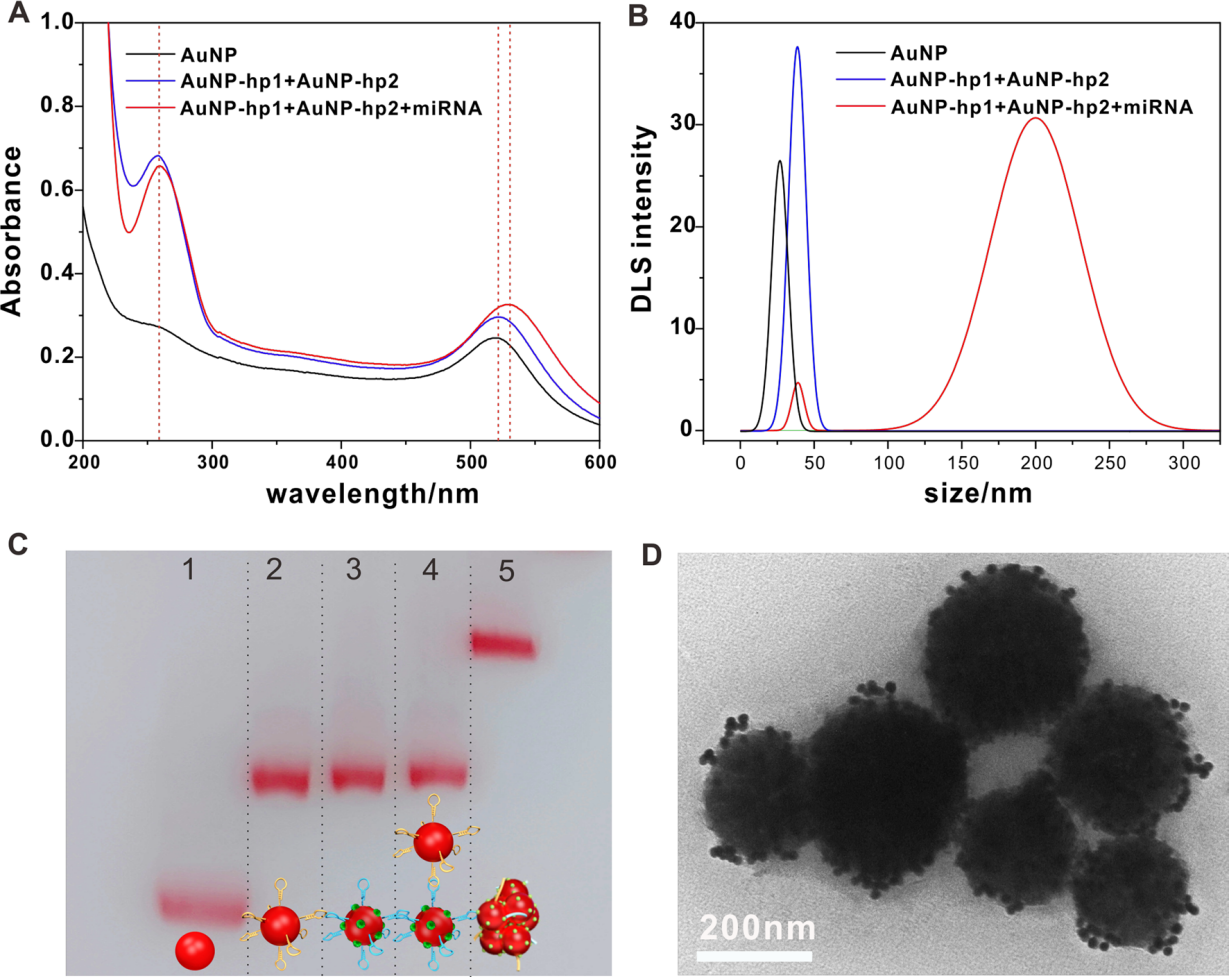
Characterization of AuNP aggregation induced by HCR. **A** UV–Vis absorption spectra of AuNPs: a mixture of AuNPs-hp1 and AuNPs-hp2, and miRNA with AuNPs-hp1 and AuNPs-hp. **B** Dynamic light scattering spectra of AuNPs: a mixture of AuNPs-hp1 and AuNPs-hp2, and miRNA with AuNPs-hp1 and AuNPs-hp. **C** Agarose gel electrophoresis, Lanes 1–5: AuNP, AuNP-hp1, AuNP-hp2, AuNP-hp1 + AuNP-hp2, and AuNP-hp1 + AuNP-hp2 + miRNA, respectively. **D** TEM image of the self-grown products

To verify that the aggregation of AuNPs was induced by HCR, we carried out a variety of methods to characterize the products. Because the magnetic beads can cause interference, we used the enzymatic digestion products to directly trigger the reaction after demonstrating that the cleavage of DNAzyme was successful (Additional file 1: Figure S2A). The HCR sequences were also designed and verified by native PAGE analysis (Additional file 1: Figure S2B). As shown in Fig. 1A, the positive absorption band near 260 nm assigned to DNA verified that the well-designed hairpin DNA for HCR was successfully immobilized onto AuNPs [43]. The obviously increased hydration radius of AuNPs in the dynamic light scattering spectra of a mixture of AuNPs-hp1 and AuNPs-hp2 compared to that of AuNPs also indicated the successful modification of hairpin DNA on AuNPs (Fig. 1B). When we compared the UV–Vis absorption spectra of AuNPs, a mixture of AuNPs-hp1 and AuNPs-hp2, and miRNA with AuNPs-hp1 and AuNPs-hp (shown in Fig. 1A), the absorption band assigned to AuNP shifted from 520 to 530 nm after adding target miRNA to the mixture of AuNPs-hp1 and AuNPs-hp2; this red shift indicated that aggregates were formed by the enzymatic digestion products [44]. In addition, the new complete peak that appeared in the dynamic light scattering spectra of the miRNA with AuNPs-hp1 and AuNPs-hp also indicated that large aggregates were formed in the presence of target miRNA (Fig. 1B) [45]. As shown in Fig. 1C, agarose gel electrophoresis showed that the band migrated significantly more slowly after the HCR trigger chain was added (lanes 4–5), which may have been caused by the combination of gold nanotags [46]. Furthermore, we executed the entire process on magnetic nanoparticles, and we prepared the samples of the final products for TEM analysis by washing most of the salt out of the samples. Although some aggregates may have fallen off during the TEM sample preparation process, many gold nano-aggregates still appeared on the surface of the magnetic particles (Fig. 1D). Compared with the smooth surface of magnetic nanoparticles with AuNP-hp1 without miRNA, there were many aggregates on the surface of the magnetic nanoparticles in the presence of target miRNA, AuNP-hp1, and AuNP-hp2 (Fig. 1D vs. Additional file 1: Figure S4A). The appearance of magnetic nanoparticles with miRNA, AuNP-hp1, and AuNP-hp2 was also significantly different from that of the sample without AuNP-hp2 (Fig. 1D vs. Additional file 1: Figure S4B). It is difficult to find aggregates of more than three gold particles on the surface of magnetic beads without HCR (Additional file 1: Figure S4B). The above characterization proved that the HCR and nano-aggregation were successfully integrated, and with the help of the corresponding DNAzyme probe, this strategy could be employed in circulating miRNA detection.

### Analysis of circulating miRNAs by the SERS platform

**Fig. 2:**
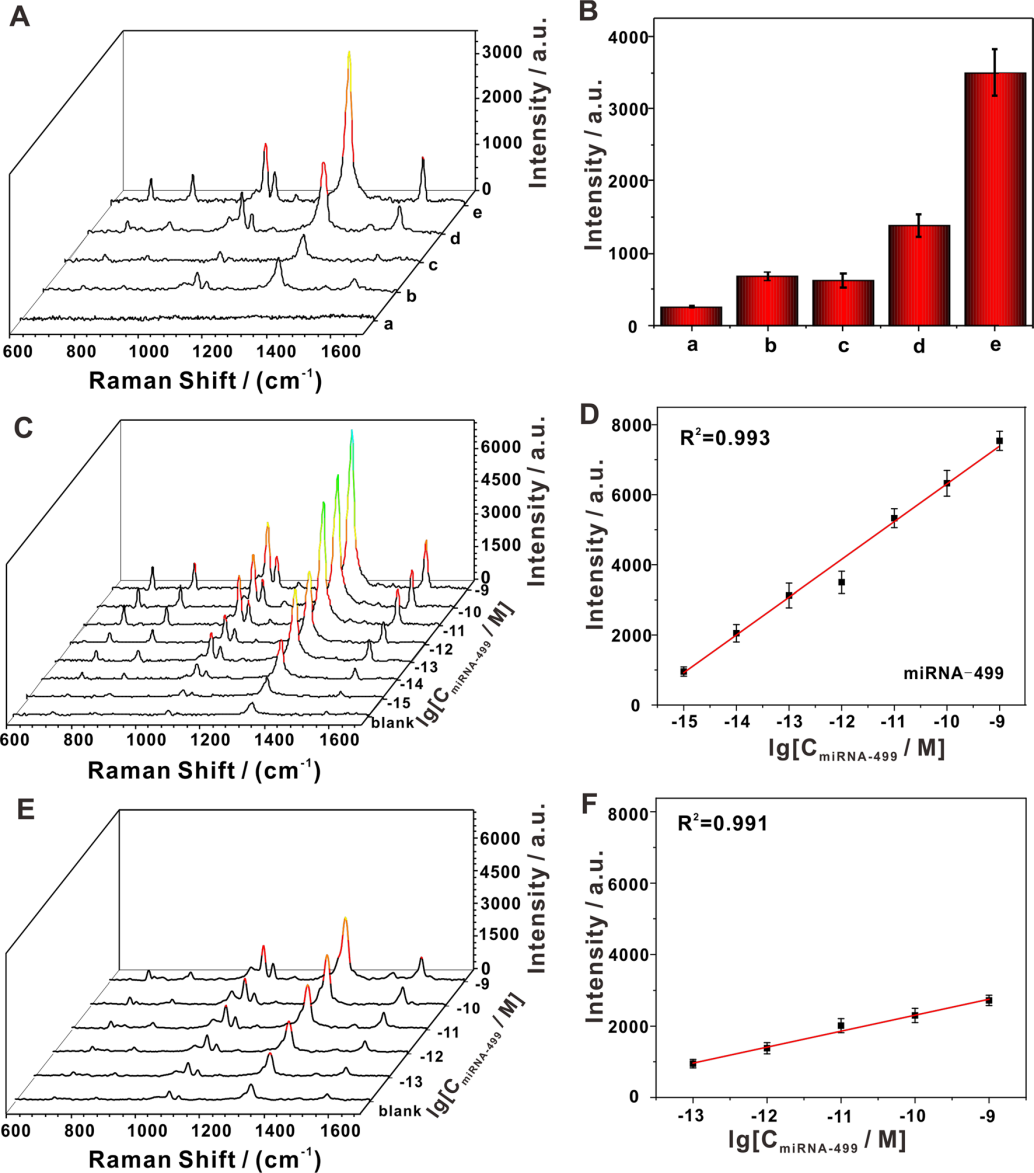
Comparison of performance of the developed SERS with the agglomeration-free system. **A** SERS spectra of different control groups and **B** the corresponding SERS intensity at 1340 cm^−1^. (a) MBP, (b) MBP + AuNP-hp1, (c)MBP + AuNP-hp1 + AuNP-hp2, (d)MBP + AuNP-hp1 + miRNA, and (e) MBP + AuNP-hp1 + AuNP-hp2 + miRNA; the concentration of miRNA-499 used was 1 pM. **C** Representative SERS spectra collected by the present platform in the presence of miRNA-499 at various concentrations. **D** A standard curve constructed based on the intensity of the 1340 cm^−1^ peak obtained from miRNA-499 detection by the present platform. **E** Representative SERS spectra collected by the agglomeration-free system in the presence of miRNA-499 at various concentrations. **F** Linear regression based upon the intensity of the 1340 cm^−1^ peak obtained from miRNA-499 detection by the agglomeration-free system

The detection principle includes target recognition and signal amplification, factors involved in both components that should be considered for SERS measurement. Target recognition was realized by a composite DNAzyme structure, which cleaved the rA site on the magnetic biological probe (MBP). Then the cleavage product opened hp 1 on the AuNP-hp 1 reporter and initiated the HCR to bind large amounts of AuNP-hp 1 and AuNP-hp 2. After comprehensive consideration, we studied the influence of MBP, target miRNA, AuNP-hp 1 reporter and AuNP-hp 2 reporter on the SERS signal. Figure 2A shows the SERS spectra obtained from different control experiments, and Fig. 2B shows the comparison of the Raman intensity at 1340 cm^−1^ for each corresponding control group. As all the Raman signals were derived from gold nanotags, the shape of each Raman spectrum in the different control groups was similar (Fig. 2A). Since probe DNA on the MBP and hp1 on the AuNP produce a small amount of by-product double chain, a certain background signal was observed in the SERS spectra of sample b and sample d; and a small number of individual AuNPs in Fig. 1D also partially explains the background signal source. Upon the addition of miRNA-499, the DNAzyme was activated; as a result, the product on the probe directly hybridized with the first gold nanotag to produce a peak that possessed a significantly enhanced signal compared to the background signal. After the second gold nanotag was added, the SERS signal was further enhanced; this indicated that the HCR-induced nano-aggregation augmented the SERS signal, and thus improved overall detection performance. The above results indicated that our design was reasonable and that our platform could be used to detect circulating miRNA.

To investigate the performance of our developed SERS sensing platform, it was applied to analyze circulating miRNAs. Prior to the SERS measurements, key factors influencing the reaction such as ion concentration and reaction time were optimized to obtain the best SERS performance. As shown in Additional file 1: Figure S3, the optimal concentration of Mg^2+^ was 1o mM, and 2.5 h was enough for HCR and DNAzyme requires 9 h to achieve the maximal cutting effect. Subsequently, we explored the sensitivity and the linear detection range of the proposed biosensor, and found that the lowest detectable concentration (LDC) with regards to miRNA-499 concentration of our platform was 1 fM; and that the detectable linear range was between 1 fM and 1 nM, with a limit of detection (LOD) of 0.37 fM (Fig. 2C and D). We further investigated the effect of HCR-induced aggregation on the quantitative detection capability of our developed SERS sensing platform by comparing the detection limits and the linear ranges with those of the agglomeration-free system. As shown in Fig. 2E, the biosensor without HCR-induced aggregation was applied to detect miRNA-499 at varying concentrations, and miRNA-499 at a concentration of 1 fM caused distinguishable changes in the SRES signal, indicating that the LDC with DNAzyme alone was 100 fM [47]. As shown in Fig. 2F, from the plot of SERS intensity vs. logarithmic concentration of miRNA-499, we concluded that the DNAzyme-assisted biosensor was linear from 100 fM to 1 nM, and we calculated its LOD to be 87 fM (S/N = 3). The comparison results clearly showed that the aggregation caused by HCR further improved the sensitivity of our SERS sensor. Our platform thus displayed both an improved detection limit and a wider linear range in the analysis of circulating miRNAs.

**Fig. 3:**
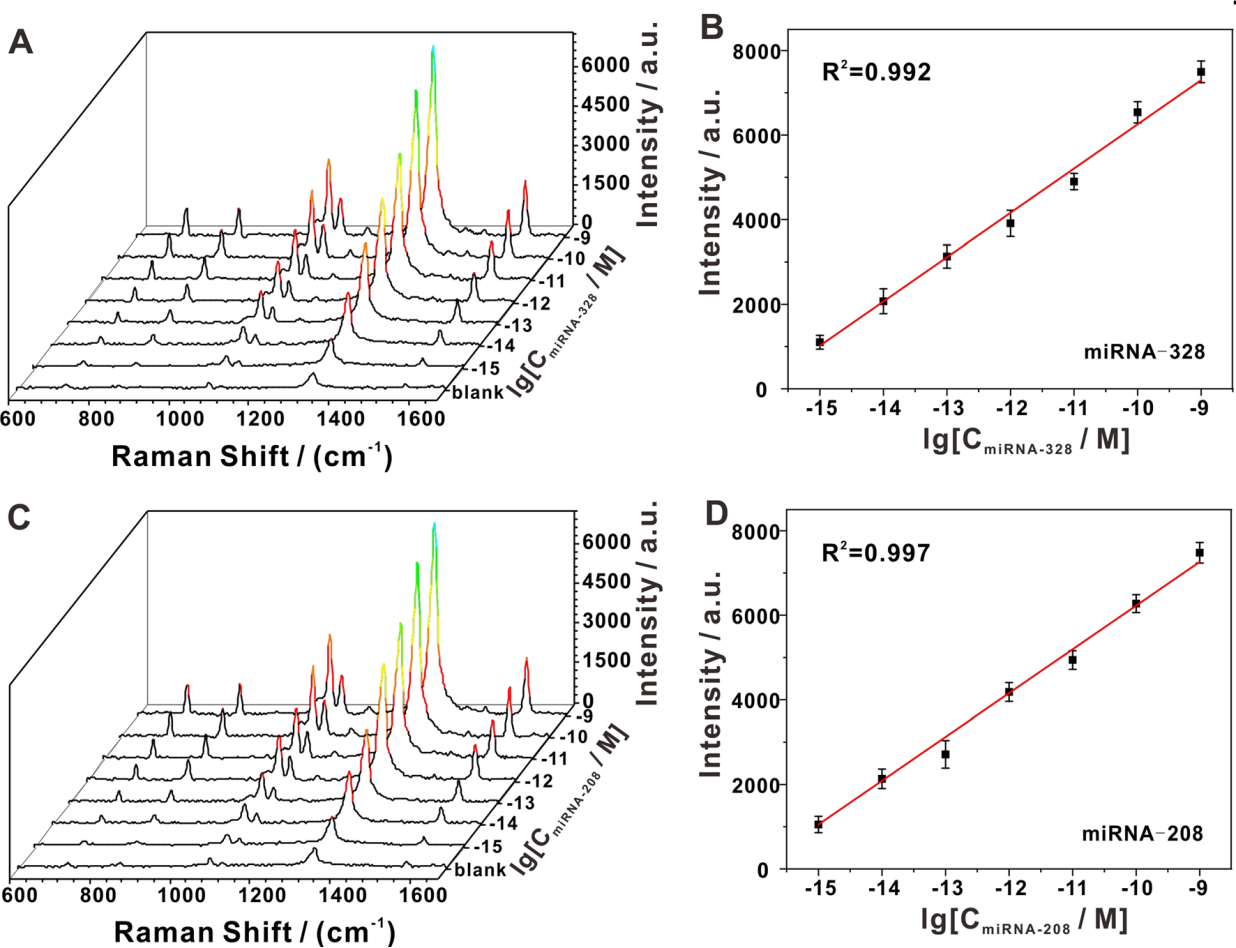
Quantitative analysis of different miRNAs. **A** Representative SERS spectra of miRNA-328 at various concentrations. **B** Linear regression constructed using the 1340 cm^−1^ peak intensity obtained from miRNA-328 detection. **C** Representative SERS spectra of miRNA-208 at various concentrations. **D** A standard curve constructed using the 1340 cm^−1^ peak intensity obtained from miRNA-208 detection

To evaluate the versatility of our platform, with respect to its capacity in high-throughput clinical analysis, two randomly selected circulating miRNAs (miRNA-328 and miRNA-208) were analyzed. According to the SERS spectra, we noted that 1 fM of the two circulating miRNAs caused distinguishable signal changes (Fig. 3A and C). Additionally, we observed an excellent linear relationship between the SERS intensity at 1340 cm^−1^ and the logarithmic concentration of circulating miRNA from 1 fM to 1 nM (Fig. 3B and D). The calculated LODs for miRNA-328 and miRNA-208 were 0.47 fM (S/N = 3) and 0.56 fM (S/N = 3), respectively, indicating that our SERS platform manifested similar detection sensitivities toward different circulating miRNAs, and thus could be applied to detect a variety of circulating miRNAs. In addition, we compared the detection capacity of our SERS platform with that of other SERS biosensors in detecting circulating miRNAs, and our results revealed that the detection limit and quantitative range of our platform were superior to most other SERS biosensors without the need for complex substrates (Additional file 1: Table S2).

**Fig. 4:**
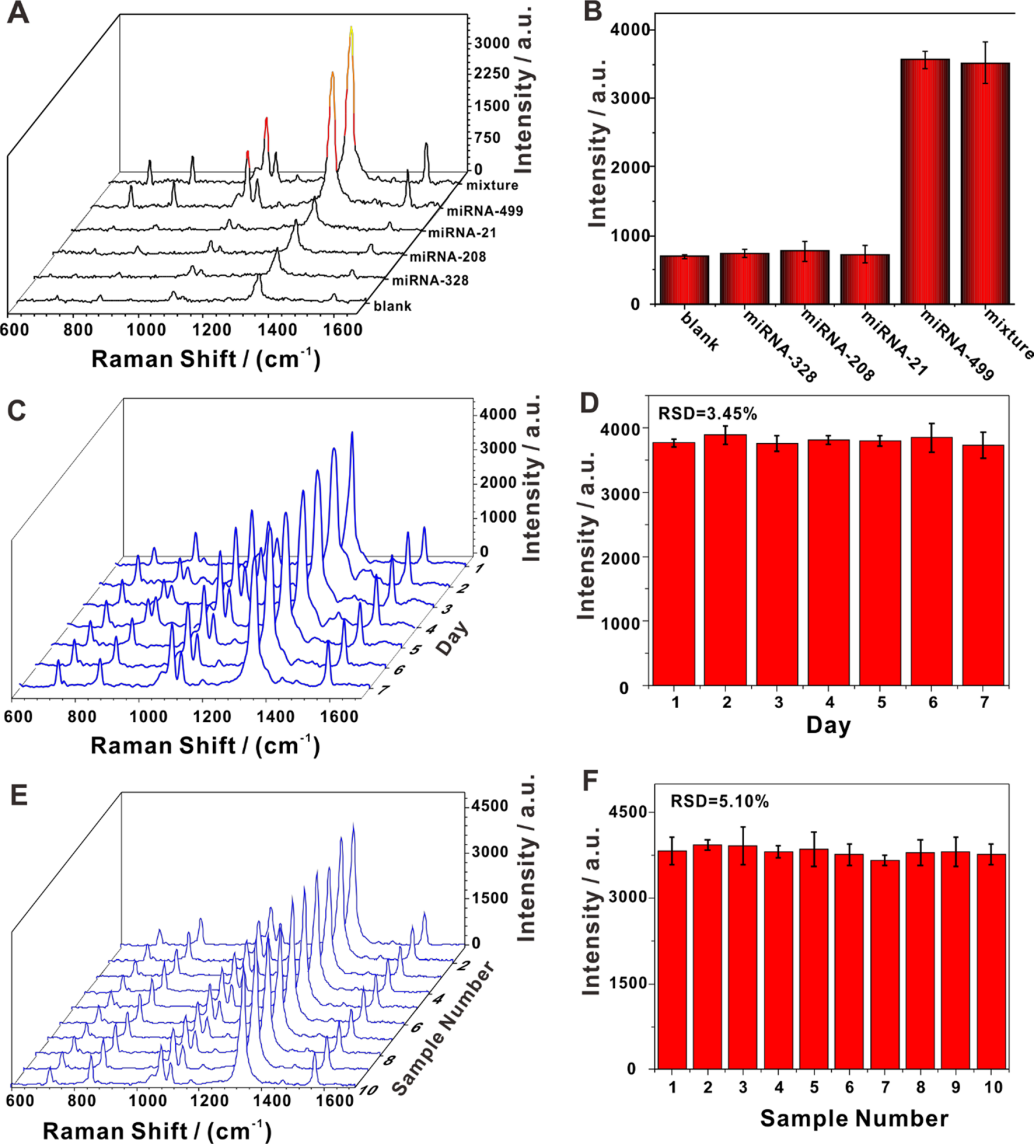
Evaluation of selectivity, stability, and reproducibility of the developed SERS platform. **A** Representative SERS spectra and **B** mean SERS intensity obtained from the detection of miRNA-499, miRNA-328, miRNA-208, miRNA-21 and their mixture. **C** SERS spectra and **D** Raman intensities obtained from 7 days of the same sample. **E** SERS spectra and **F** Raman intensities obtained from 10 different biological samples over 10 days. All of the measurements were performed at a miRNA concentration of 1 pM. Data depicted in **B**, **D**, and **F** are the intensities of the peaks at 1340 cm^−1^

Specificity is an important criterion used to determine the reliability of biosensors. Due to high sequence identity among circulating miRNAs, specificity has become a challenging problem facing the use of biosensors [48]. Tests on the specificity for miRNA-499 of our sensing platform were carried out using three non-complementary circulating miRNAs (miRNA-328, miRNA-208, and miRNA-21). As can be seen in Fig. 4A, the SERS spectra showed that miRNA-499 and the mixed miRNA sample generated significantly enhanced Raman signals, and that the intensities of the signals were similar. A comparison of the SERS intensity at 1340 cm^−1^, demonstrated that this biosensor could overcome interference by other circulating miRNAs. Thus, it was specific to the target miRNA (Fig. 4B). These results successfully revealed that our SERS biosensor was highly specific to the target miRNA.

Stability and reproducibility are also essential criteria for assessing SERS biosensor performance [49]. To investigate the stability of our SERS platform, the same sample was scanned over 7 days, and the obtained spectra showed high consistency with an RSD of 3.45% (Fig. 4C and D), indicating that the platform had good stability and high reliability. Moreover, the data from 10 different biological samples over 10 days showed an RSD of 5.10%, revealing that the proposed SERS biosensor possessed both technical and biological reproducibility (Fig. 4E and F) [50]. These results indicated that our sensor platform was stable and reliable, that it exhibited high sensitivity, and that it could potentially be used for the practical detection of circulating miRNAs.

### Detection of serum samples by the SERS platform

**Fig. 5:**
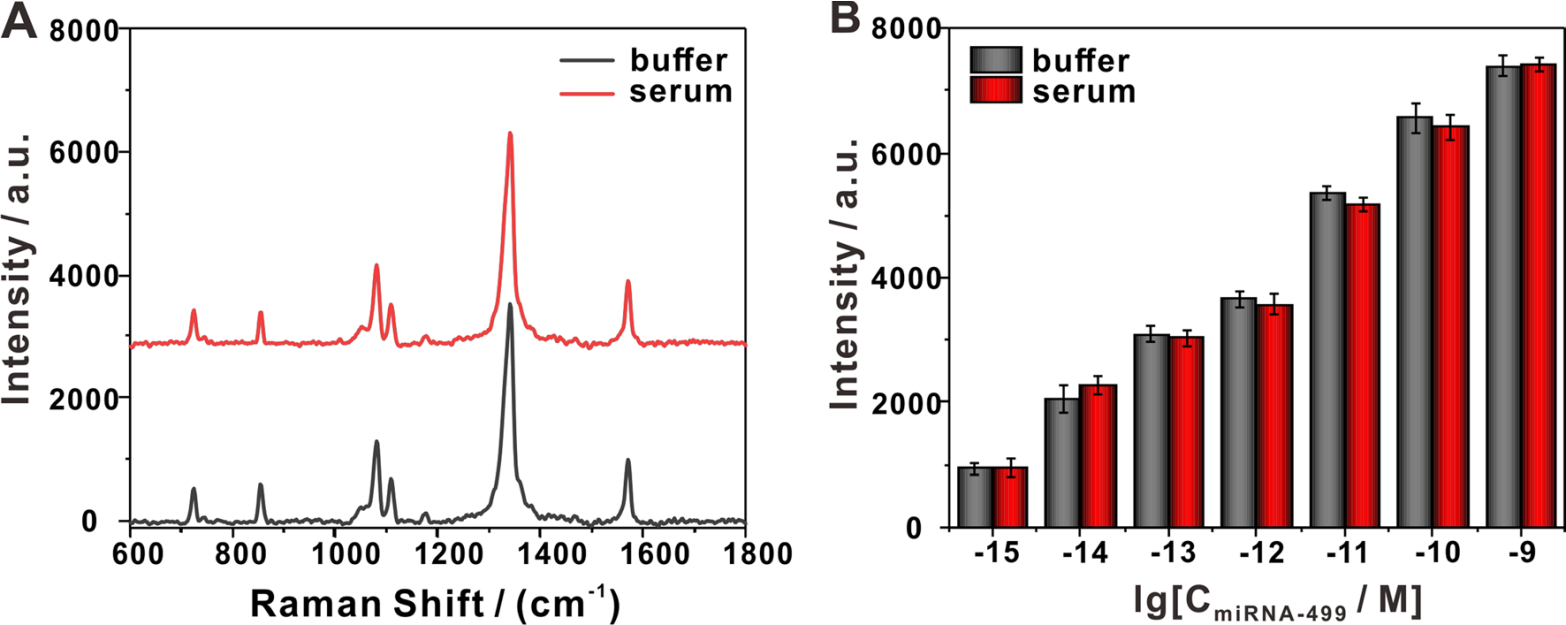
Applicability of detection. **A** Representative SERS spectra obtained from the detection of 1 pM miRNA-499 in TE buffer and in serum. **B** Comparison of SERS peak intensities at 1340 cm^−1^ of miRNA-499 at different concentrations in TE buffer and in serum

Although its detection limits did not exceed those of some extreme cases, the platform may still hold great potential in clinical applications due to its high sensitivity and reliability. To explore its practicality, the proposed SERS sensor was employed to detect circulating miRNA in serum with a proof-of-principle approach using spiked miRNA-499. There was only a very low background signal in the serum without miRNA-499, which did not affect the quantitative detection (Additional file 1: Figure S5). As shown in Fig. 5A, the SERS spectra obtained from the detection of serum samples were consistent with those obtained from the buffer, indicating that this platform overcome interferences by substances in serum. As shown in Fig. 5B, further quantitative analysis showed that the results of the serum samples were not significantly different from those from the buffer, which further confirmed our perception that our biosensor performed well in serum. The above results revealed that our multi-level amplified SERS sensing platform detected the target analytes in serum samples, and thus may yield broad practical applications.

## Conclusions

For the present study, we designed a reliable SERS sensing platform to detect circulating miRNAs by integrating nucleic acid amplification with nanoparticle aggregation. By efficient identification and amplification of DNAzyme, numerous HCR triggers were released to promote the aggregation of nanoparticles, which greatly improved the performance of the SERS platform. The sensitivity of the SERS platform could also be improved with respect to the increase of the number of hot spots. In detecting multiple circulating miRNAs, the platform exhibited the lowest detectable concentration of 1 fM and a linear range between 1 fM and 10 nM, which indicates that were superior to those of the widely used sandwich-type SERS detection. Due to the influence of steric hindrance and the washing process, our platform may not be applicable for some extreme cases. Nonetheless, it is obvious that our platform can be employed to detect circulating miRNAs and it may potentially be developed to become a high-throughput clinical detection method.

## Supplementary Information

**Additional file 1:** **Figure S1.** The custom-made cuvette and detection device of the portable Raman system (BWTEK). **Figure S2.** Native PAGE analysis of the DNAzyme composite structure and the cascade amplification process. **Figure S3.** Optimization of key factors for the reaction. **Figure S4.** TEM image of the magnetic beads with AuNP-hp1 without miRNA, and with miRNA. **Figure S5.** The SERS spectra of serum without the addition of miRNA-499. **Figure S6.** Comparison of SERS performance of this strategy with 16 nm AuNPs and 30 nm AuNPs. **Table S1.** Sequences of all oligonucleotide used in this study. **Table S2.** Comparison of biosensors for the detection of AMI-related miRNAs

## Data Availability

All data are included in this published article.
