# Supplementary material for: Multistage nucleic acid amplification induced nano-aggregation for 3D hotspots-improved SERS detection of circulating miRNAs
Source: J Nanobiotechnology. 2022 Jun 16;20:285. doi: 10.1186/s12951-022-01500-y (PMC9205088; doi:10.1186/s12951-022-01500-y)
Supplement: Supplementary file 1 — Additional file 1: Figure S1. The custom-made cuvette and detection device of the portable Raman system (BWTEK). Figure S2. Native PAGE analysis of the DNAzyme composite structure and the cascade amplification process. Figure S3. Optimization of key factors for the reaction. Figure S4. TEM image of the magnetic beads with AuNP-hp1 without miRNA, and with miRNA. Figure S5. The SERS spectra of serum without the addition of miRNA-499. Figure S6. Comparison of SERS performance of this strategy with 16 nm AuNPs and 30 nm AuNPs. Table S1. Sequences of all oligonucleotide used in this study. Table S2. Comparison of biosensors for the detection of AMI-related miRNAs [file 12951_2022_1500_MOESM1_ESM.docx]

Supporting Information

**Multistage Nucleic Acid Amplification Induced Nano-Aggregation for 3D Hotspots-Improved SERS Detection of Circulating miRNAs**

*Yudie Sun^1^, La Fang^1^, Yang Yi^1^, Aobo Feng^1^, Kui Zhang^1^* and Jing-Juan Xu^2^*

^1^ School of Chemistry and Chemical Engineering, Anhui University of Technology, Ma Xiang Road, Ma ‘anshan, Anhui 243032, P. R. China; E-mail:  [zhangkui@mail.ustc.edu.cn](mailto:%20o@ustc.edu.cn)

^2^ State Key Laboratory of Analytical Chemistry for Life Science, School of Chemistry and Chemical Engineering, Nanjing University, Nanjing 210023, P. R. China.

Contents

**Additional file 1: Figures**.

Figure S1. The custom-made cuvette and detection device of the portable Raman system (BWTEK).

Figure S2. Native PAGE analysis of the DNAzyme composite structure and the cascade amplification process.

Figure S3. optimization of key factors for the reaction.

Figure S4. TEM image of the magnetic beads with AuNP-hp1 without miRNA, and with miRNA.

Figure S5. The SERS spectra of serum without the addition of miRNA-499.

Figure S6. Comparison of SERS performance of this strategy with 16 nm AuNPs and 30 nm AuNPs.

**Additional file 1: Tables**

Table S1. Sequences of all oligonucleotide used in this study.

Table S2. Comparison of biosensors for the detection of AMI-related miRNAs.

**·References**

**Additional file 1: Figures**


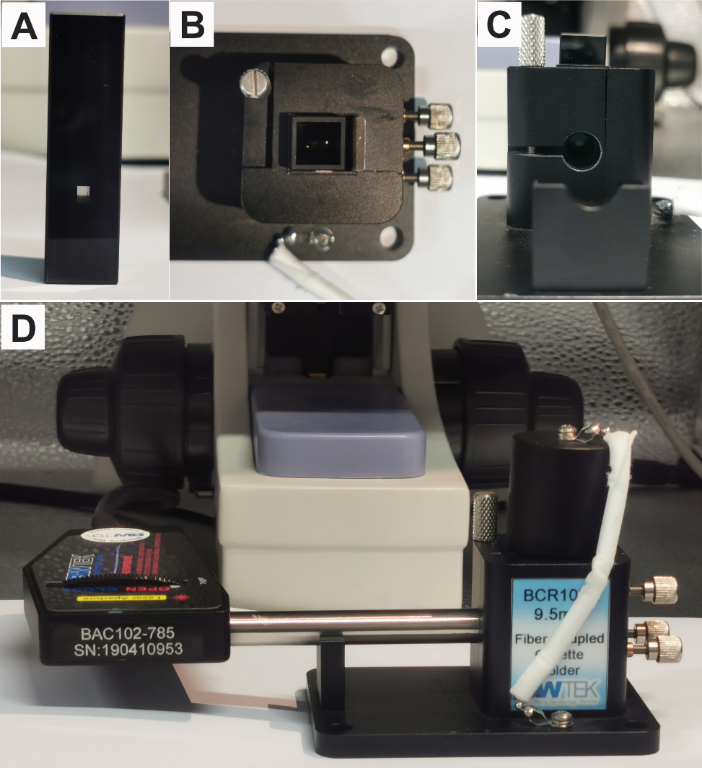


Figure S1. The custom-made cuvette and detection device of the portable Raman system (BWTEK).


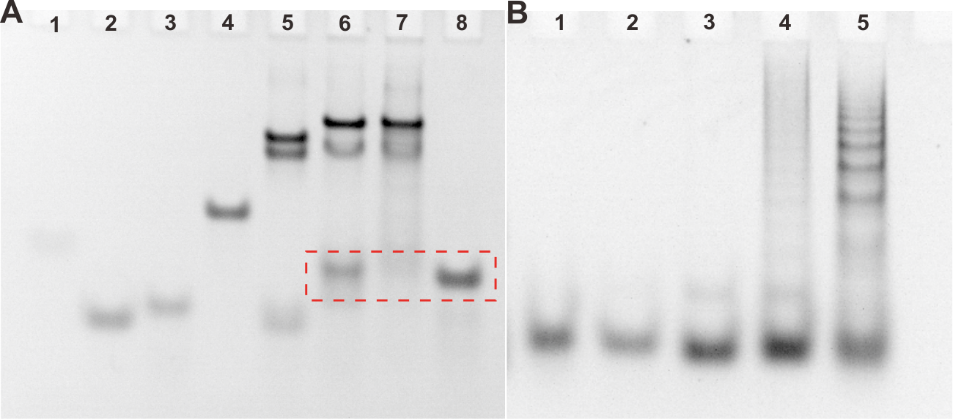


Figure S2. Native PAGE analysis of the DNAzyme composite structure and the cascade amplification process. (A) 10% PAGE analysis of DNAzyme. Lane 1) target miRNA, 2) DA, 3) DB, 4) target miRNA + DA +DB, 5) DA + DB + probe, 6) target miRNA + DA +DB+probe (with rA site), 7) target miRNA + DA + DB + probe (without rA site), 8) DNAzyme-digested product. (B) 6% PAGE analysis of the cascade amplification process. Lanes 1-5: DNAzyme-digested product, hp1, hp2, hp1+hp2, DNAzyme-digested product + hp1 + hp2.


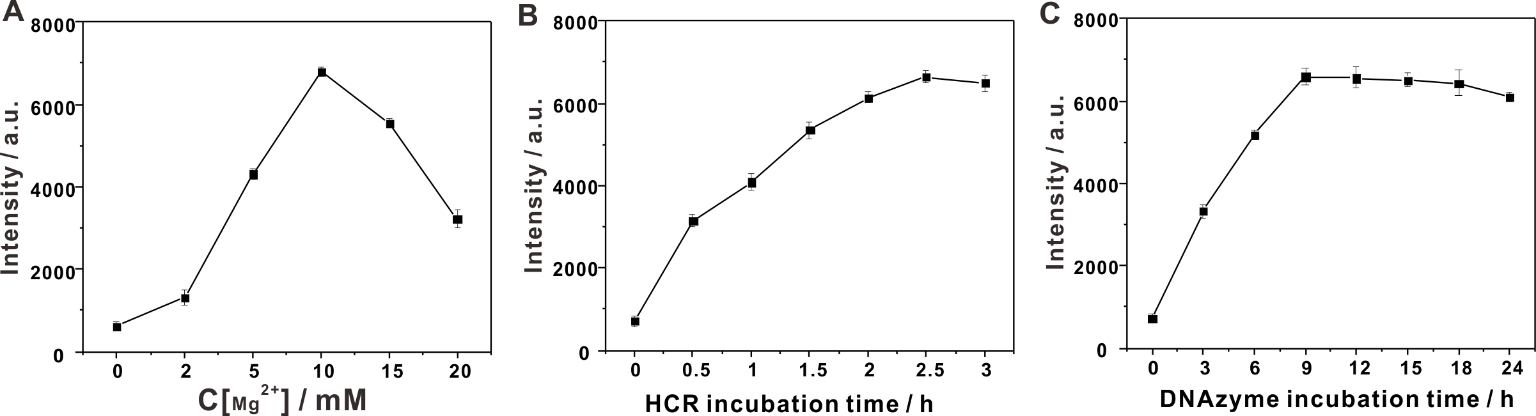


Figure S3. Effects of (A) Mg^2+^ concentration, (B) HCR incubation time, and (C) enzymatic time on the change of the SERS signal at 1340 cm^−1^. 0.10 nM of miRNA-499 was used here.

**
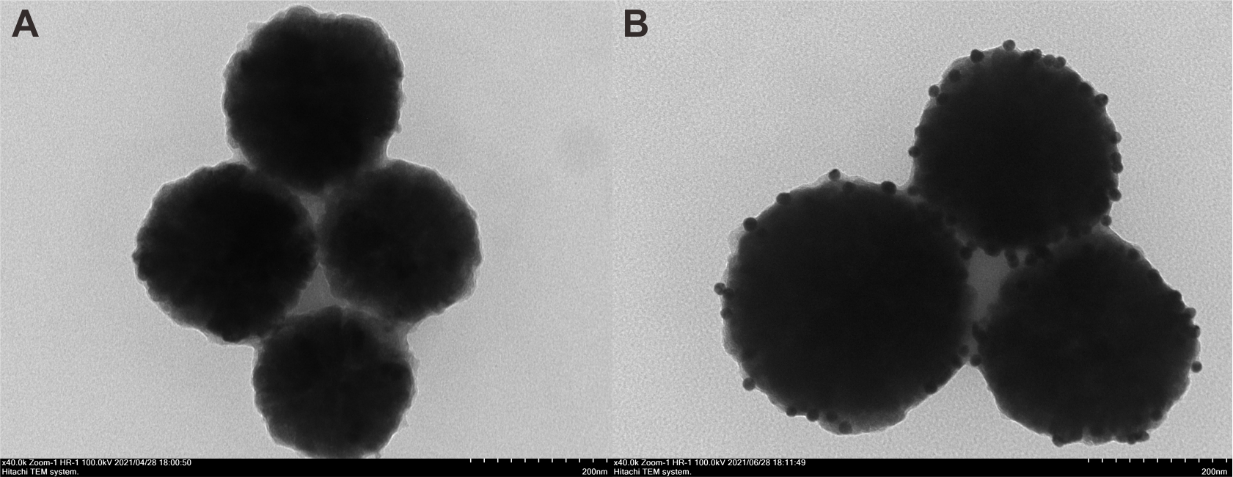
**

Figure S4. TEM image of the magnetic beads with AuNP-hp1 (A) without miRNA, and (B) with miRNA.

**
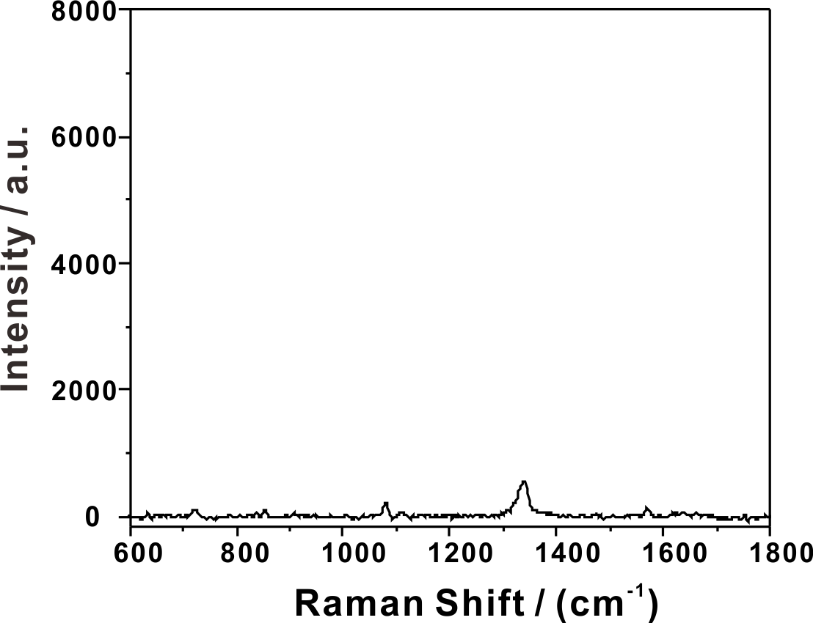
**

Figure S5. The SERS spectra of serum without the addition of miRNA-499.


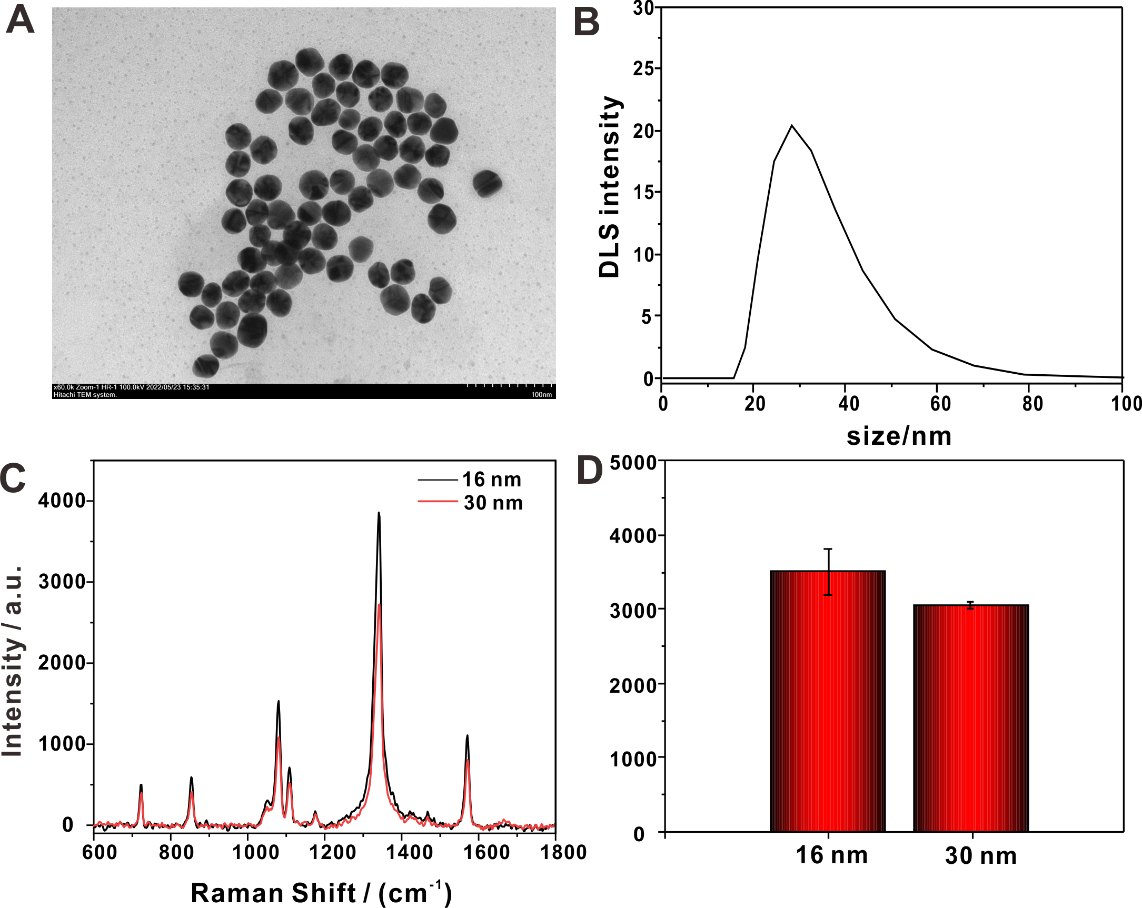


Figure S6. TEM image (A) and Dynamic light scattering spectra (B) of 30 nm AuNPs. Representative SERS spectra (C) and mean SERS intensity (D) obtained from the detection of miRNA-499 (1 pM) with 16 nm AuNPs and 30 nm AuNPs.

**Additional file 1: Tables**

**Table S1. Sequences of Oligonucleotide Used in This Study.**

| **Name** | **Sequence (5’-3’)** |
| --- | --- |
| probe | CTGATAAGCTACAGGACATCGAATAGTCTTTTTTGAGCGACACACTATrAGGAAGAGATACTTTTTTGACTATTCGA |
| Hp1 | SH-(CH_2_)_6_-TTTTTTTTTTTTTTTTTTTCATCGAATAGTCCTGACTGACTATTCGATGTCCTGT |
| Hp2 | AGTCAGGACTATTCGATGACAGGACATCGAATAGTCTTTTTTTTTTTTTTTTTTT-(CH_2_)_6_-SH- |
| miRNA-499 | UUAAGACUUGCAGUGAUGUUU |
| DA(499) | GTATCTCTTCCGCGATTAACCAAGTCTTAA |
| DB(499) | AAACATCACTGGTTAGACCCATGTTAGTGTGTCGCTC |
| miRNA-328 | CUGGCCCUCUCUGCCCUUCCGU |
| DA(328) | GTATCTCTTCCGCGATTAACCAGAGGGCCAG |
| DB(328) | ACGGAAGGGCAGTTAGACCCATGTTAGTGTGTCGCTC |
| miRNA-208 | AAGCUUUUUGCUCGAAUUAUGU |
| DA(208) | GTATCTCTTCCGCGATTAACAGCAAAAAGCTT |
| DB(208) | ACATAATTCGGTTAGACCCATGTTAGTGTGTCGCTC |
| trigger | CTGATAAGCTACAGGACATCGAATAGTCTTTTTTGAGCGACACACTAT |

**Table S2. Comparison of SERS biosensors for the Detection of miRNAs in human serum.**

| **Analytical method** | **nucleic acid amplification** | **Target** | **Linear range** | **LOD** | **substrate** | **Ref.** |
| --- | --- | --- | --- | --- | --- | --- |
| SERS | catalytic hairpin assembly | miRNA-196a-5p& miRNA-31a-5p | 0.1 fM-1.0 pM | 1.681 nM | Au–Ag nanoshuttles | S^1^ |
| SERS | (DSN)-assisted signal amplifica-  tion | miRNA-21 | 12 fM-18 pM | 5 fM | Au@R6G@AuAg nanoparticles | S^2^ |
| SERS | no | miRNA-21, miRNA-141, miRNA-31 | 10fM-100pM | 10 fM | gold−silver nanomushroom | S^3^ |
| SERS | Catalytic Hairpin Self-Assembly | miR-1246, miR-221, miR-133a, and miR-21 | 0.50 pM-100 nM | 0.15 pM | AuAgNP | S^4^ |
| SERS | no | miRNA-133a and miRNA-499 | 1 fM-100 pM | 393 aM | Ag nanorod array | S^5^ |
| SERS | catalytic hairpin assembly | miRNA-499 | 1fM-10nM | 0.306 fM | Ag/Au nanosphere | S^6^ |
| SERS | no | miRNA-21 | 10fM-100nM | 0.839fM | gold−silver nanosnowmen | S^7^ |
| SERS | no | miRNA-141 | four orders of magnitude | 120fM | DSNB-SA@GNPs | S^8^ |
| SERS | HCR | microRNA-21 | 1fM-10pM | 0.3fM | AgNP | S^9^ |
| SERS | DNAzyme & HCR | miRNA-499, miRNA-208, miRNA-328 | 1fM-10nM | 0.37fM | AuNP | This work |

**References**

(1) Cao, X.; Sun, Y.; Mao, Y.; Ran, M.; Liu, Y.; Lu, D.; Bi, C. Rapid and sensitive detection of dual lung cancer-associated miRNA biomarkers by a novel SERS-LFA strip coupling with catalytic hairpin assembly signal amplification. *J. Mater. Chem. C* **2021**, *9*, 3661-3671.

(2) Ma, D.; Huang, C.; Zheng, J.; Tang, J.; Li, J.; Yang, J.; Yang, R. Quantitative detection of exosomal microRNA extracted from human blood based on surface-enhanced Raman scattering. *Biosens. Bioelectron.* **2018**, *101*, 167-173.

(3) Su, J.; Wang, D.; Norbel, L.; Shen, J.; Zhao, Z.; Dou, Y.; Peng, T.; Shi, J.; Mathur, S.; Fan, C., et al. Multicolor Gold-Silver Nano-Mushrooms as Ready-to-Use SERS Probes for Ultrasensitive and Multiplex DNA/miRNA Detection. *Anal. Chem.* **2017**, *89*, 2531-2538.

(4) Si, Y.; Xu, L.; Deng, T.; Zheng, J.; Li, J. Catalytic Hairpin Self-Assembly-Based SERS Sensor Array for the Simultaneous Measurement of Multiple Cancer-Associated miRNAs. *ACS Sens.* **2020**, *5*, 4009-4016.

(5) Song, C. Y.; Yang, Y. J.; Yang, B. Y.; Sun, Y. Z.; Zhao, Y. P.; Wang, L. H. An ultrasensitive SERS sensor for simultaneous detection of multiple cancer-related miRNAs. *Nanoscale* **2016**, *8*, 17365-17373.

(6) Sun, Y.; Li, T. Composition-Tunable Hollow Au/Ag SERS Nanoprobes Coupled with Target-Catalyzed Hairpin Assembly for Triple-Amplification Detection of miRNA. *Anal. Chem.* **2018**, *90*, 11614-11621.

(7) Guo, R.; Yin, F.; Sun, Y.; Mi, L.; Shi, L.; Tian, Z.; Li, T. Ultrasensitive Simultaneous Detection of Multiplex Disease-Related Nucleic Acids Using Double-Enhanced Surface-Enhanced Raman Scattering Nanosensors. *ACS Appl. Mater. Interfaces* **2018**, *10*, 25770-25778.

(8) Zhang, H.; Fu, C.; Wu, S.; Shen, Y.; Zhou, C.; Neng, J.; Yi, Y.; Jin, Y.; Zhu, Y. Magnetic-capture-based SERS detection of multiple serum microRNA biomarkers for cancer diagnosis. *Analytical Methods* **2019**, *11*, 783-793.

(9) Zheng, J.; Ma, D.; Shi, M.; Bai, J.; Li, Y.; Yang, J.; Yang, R. A new enzyme-free quadratic SERS signal amplification approach for circulating microRNA detection in human serum. *Chem. Commun.* **2015**, *51*, 16271-16274.
